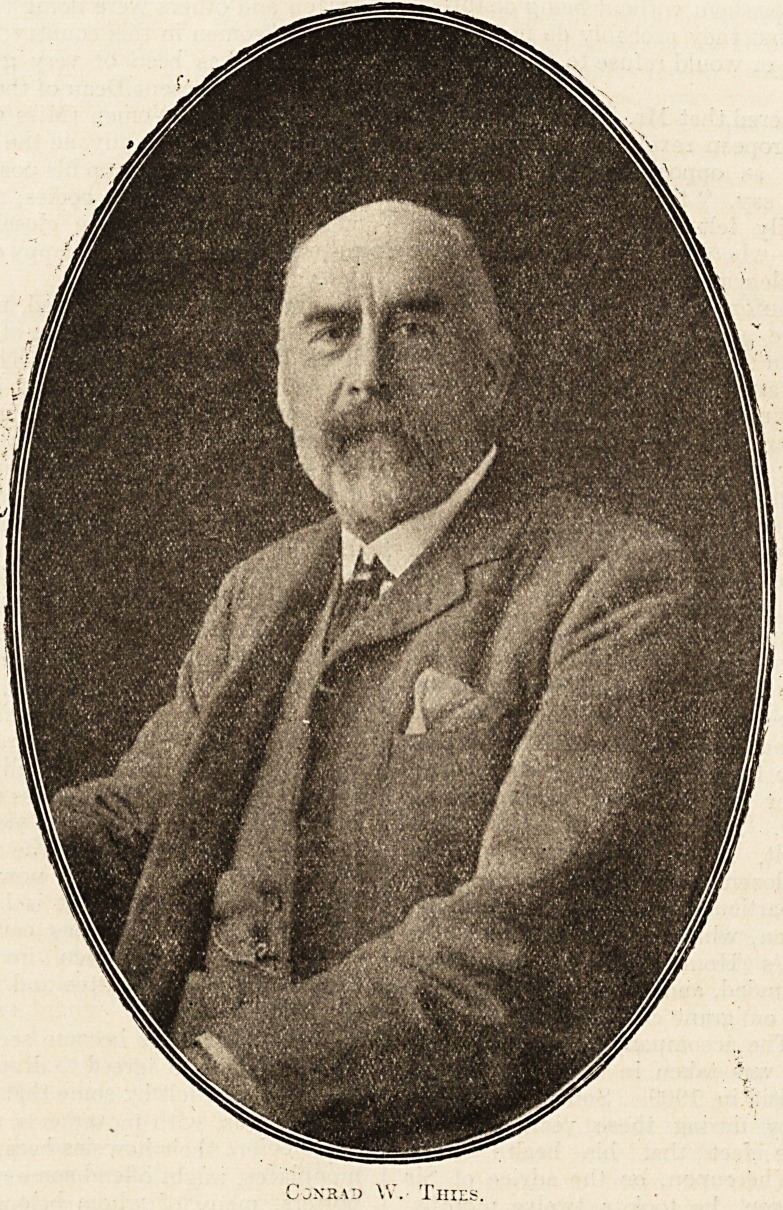# A Hospital Officer's Career

**Published:** 1911-08-05

**Authors:** 


### A HOSPITAL OFFICER'S CAREER.

### MR. THIES' RECORD AT THE ROYAL FREE HOSPITAL.

With August the last month of Mr. Conrad W. Thies' twenty-four years' service as secretary to the Royal Free Hospital begins. Only last week, at the meeting of the Committee on July 26, Sir Edwin Durning-Lawrence moved, and Mr. Holroyd Chaplin seconded, a farewell resolution in acknowledgment of his achievement, signed by the President (H.R.H. Princess Christian), by Lord Sandwich, Chairman of the Committee, and by Mr. Chaplin, Chairman of the Weekly Board, which will be engrossed and sealed for presentation to Mr. Thies at a later date. In the meantime it becomes of interest to cast a retrospective glance over Mr. Thies' career, and to sum up rapidly how it was that he first became attracted to hospital work and what he has done for the Royal Free Hospital.

Conrad W. Thies.

Mr. Thies is a born cockney, for the local registrar records that he first opened his eyes in Bow Lane in 1848. City born, he was city educated at the ancient foundation of Christ's Hospital, to which he was sent at the early age of seven and at which he remained till he was fourteen years old. He then had to start earning his own living, which he proceeded to do by obtaining employment with Messrs. Tapling and Company, wholesale warehousemen. With them he remained for twenty-two years, during which he worked through most of the departments, especially the counting-house, and, after having been employed in various capacities, he was eventually made manager of a department.

The soul of Mr. Thies, however, was not satisfied with the mere struggle for existence, for while employed by the warehousemen he spent what spare time he had—that is to say, his evenings—in organising lectures and concerts for working people, in making music accessible to them: in short, in finding out what life was like by the two good methods of theorising on its principles and of organising one or two of its needs. The first method, lecture-haunting, is probably the simplest way in which anyone can train himself for public life; and the second, philanthropic work, supplements the former by teaching one how ideas can be made to fructify, and how people can be made to do what is best for them without being disturbed by the knowledge that they probably do not understand your reasons, or would refuse to follow you if they did.

Let it be remembered that Mr. Thies was born in 1848, a year of European revolution, in which the idea of nationality as opposed to that of "the State," or, as we say, "the Government," was making itself noisily felt everywhere in Europe except in England—where, thanks to the immunity from European tendencies which Napoleon's defeat at Waterloo had unfortunately preserved, revolution took the more amiable form of philosophic radicalism. Darwin's "Origin of Species" was published in 1859 and the sentimental and somewhat pious philanthropic individualism of Maurice, Kingsley, Martineau, and others was giving place to a wave of secular thought that was shortly to express itself in the various forms of pessimistic agnosticism, women's emancipation, scientific socialism, and the æsthetic and pre-Raphaelite movements. These have developed various changes of title and content since then, but it was on them that Mr. Thies' youthful mind was nourished and among them his spiritual wild oats were sown. Thus we find him, at the second or middle period of his life, a frequenter of South Place Institute, where, or in varying degrees influenced by which, many of those now famous as economists, men of letters, Fabians or artists, were then rubbing shoulders together. Mr. Shaw, Mr. and Mrs. Webb, H. S. Salt, Edward Carpenter, Walter Crane, are half a dozen ready examples.

Mr. Thies took particular interest in the reclamation of poor children, which was carried on in the National Children's Homes that Dr. Bowman Stephenson had founded, and even went so far as to conduct parties of emigrant children to Canada in 1873 and 1875. The accompanying photograph is a snapshot which was taken in Winnipeg during Mr. Thies' last visit in 1909. Some idea of his many-sided activity during these years may be guessed from the fact that his health broke down in 1887. Whereupon, on the advice of Sir Jonathan Hutchinson, he took a twelve months' sea voyage to Australia and India. When he returned he was urged by the Right Hon. James Stansfeld and Mr. James Hopgood, both of whom were closely associated with the Royal Free Hospital, to apply for the post of secretary, which had just fallen vacant there by the death of its late holder, Mr. Blyth.

The salary offered, however, was very small, less than half of that which he had been receiving as a man of business before his health gave way, but his sympathy with the emancipation of women in general and with the cause of medical women in particular led him to apply for the post. He knew, at least, that the work would prove more congenial to him than his former business occupation had been, so his appointment was made in 1888 and he has never regretted this decision. From the early days when Dr. Garrett Anderson, the late Mrs. Isabel Thorne (see The Hospital of October 22, 1910, page 118), Miss Edith Pechey, Miss Chaplin Ayrton and others were doing the pioneer work for medical women in this country, to the present day, Mr. Thies has been of very great service to this cause. The present Dean of the Royal Free School of Medicine for Women (Miss Cock, M.D.) knows perhaps better than anyone the extent of his work. When Mr. Thies took up his post the School and the Hospital were separate bodies, not always in agreement. To-day they are closely allied and their mutual relation is a very happy one for both institutions.

As will have been expected, the past twenty-four years have seen the rebuilding of several departments of the Royal Free Hospital. These alterations cannot be catalogued here. Let it be recorded only that some £80,000 has been spent on the structure and extension of the institution. The whole of this sum has been raised by special appeals. The pathological, maternity, *x*-ray, and massage departments have been created in this period; two operating theatres have been added, and the nursing accommodation has been very much extended and improved. The raising of the capital sum which these improvements have cost has provided a very large amount of work for Mr. Thies, but while raising it by special appeals, prominent among which is the late Mr. Silver's famous notice in "Punch," he has worked extraordinarily hard and on the whole very successfully at increasing the ordinary income. The success of each appeal added automatically to the cost of maintenance, but it is stated on authority that "the financial position of the hospital is much better now than it has been at any previous period." It is by his experience in this direction that he has earned the high compliment of having been invited to become a member of the committee and weekly board of the Hospital.

When Mr. Thies became secretary of the Royal Free Hospital he agreed to drop many of his activities, as it was felt by some that a man identified, as he then was, with movements that had to wait ten years before their heresies became respectable commonplaces, might offend some of the hospital's supporters, many of whom belonged to a generation which was passing rapidly away. The result was that South Place knew him less often, but, in spite of its demands, the Royal Free Hospital did not exhaust by any means his capacity for work. The hospital world and the voluntary system now became the objects of his study. He availed himself of every opportunity to examine the changing problems which confront hospital managers, and visited in person a number of institutions in America, Canada, and on the Continent, as well as at home. Mr. Thies has written articles and read papers on hospital subjects, several of which have been published in The Hospital.

One of his particular interests has been the bringing together of hospital officers and of all those engaged in hospital administration into clubs, into societies, into associations of all sorts. He is a firm supporter of the democratic faith in the value of communal activity for all engaged in similar work. Experience has taught him that only this can cure priggishness in individuals and preserve for all the best that the individual is performing in different departments or institutions. Mr. Thies, therefore, was an active member of the Hospital Officers' Club, of which he became Hon. Secretary in 1899 and President in 1900. In 1904 he was President of the Incorporated Society of Hospital Officers. He has served on innumerable committees, and in 1890 he gave evidence before the Lords' Committee on the Metropolitan Hospitals. He was also a member of the Committee of Hospital Secretaries which formulated the present classified index of the uniform system of hospital accounts.

At the present time he is Honorary Treasurer of the British Hospitals Association, in the organisation of which he takes the greatest interest. Indeed, his last public act before leaving for a long holiday in October will be to fulfil his duties at the Conference of the Association, to be held at Manchester on September 28 and 29. It is a fitting conclusion to his success in securing adequate support for this institution that his last year of office at the Royal Free Hospital has been marked by the Silver bequest of £50,000, which crowns his secretaryship in the sense that it opens up fresh possibilities, which he bequeaths in their fulness to his successor, Mr. Garratt.

## Figures and Tables

**Figure f1:**